# Inferior Alveolar Nerve Impairment Following Third-Molar Extraction: Management of Complications and Medicolegal Considerations

**DOI:** 10.3390/jcm14072349

**Published:** 2025-03-29

**Authors:** Alessandra Putrino, Simona Zaami, Michele Cassetta, Federica Altieri, Lina De Paola, Susanna Marinelli

**Affiliations:** 1 Department of Oral and Maxillofacial Sciences, “Sapienza” University of Rome, 00161 Rome, Italy; alessandra.putrino@uniroma1.it (A.P.); michele.cassetta@uniroma1.it (M.C.); federica.altieri@aslroma1.it (F.A.); 2 Department of Anatomical, Histological, Forensic and Orthopedic Sciences, Sapienza University of Rome, 00161 Rome, Italy; lina.depaola@uniroma1.it; 3 Dental Unit, ASL Roma 1, 00161 Rome, Italy; 4 School of Law, Polytechnic University of Marche, 60121 Ancona, Italy; s.marinelli@pm.univpm.it

**Keywords:** wisdom teeth, third molars, oral surgery, maxillofacial surgery, tooth extraction, pre-operative assessment, post-operative complications, submandibular, head and neck, alveolar nerve paralysis

## Abstract

**Background:** Wisdom tooth extraction is a routine procedure with potential complications. In the lower arch, the displacement of a root or its fragment into the submandibular space is a relatively common occurrence that can lead to permanent damage to peripheral nerve fibers. Recent advancements in dental technologies, including CAD-CAM and artificial intelligence, have contributed to improved clinical outcomes in surgical procedures. **Methods:** Following a brief introductory narrative review, this clinical case describes the extraction of the left third inferior molar, which was sectioned by the oral surgeon to facilitate its removal. The procedure led to the progressive migration of a root fragment into the submandibular space, triggering an infective process. Efforts to retrieve the root fragment resulted in irreversible damage to the somatosensory motor nerves associated with the inferior alveolar nerve after the second surgery was performed by a maxillofacial surgeon. **Results:** Determining the responsibility for the damage (caused either by the oral or maxillofacial surgeon) involves both technical and ethical considerations, which are particularly relevant in cases involving re-intervention by different specialists. This case highlights the importance of a thorough preoperative evaluation of the patient’s anatomical, bone, and dental characteristics. The use of new technologies can significantly reduce the risk of complications that may otherwise lead to permanent damage and complex determinations of professional responsibility. **Conclusions:** Given the potential, albeit rare, for permanent disturbance of sensory and motor functions, managing complications and assessing the resulting damage are critical and sensitive steps in resolving such case both clinically and legally.

## 1. Introduction

Lower wisdom tooth extraction is one of the most common procedures in oral surgery. The most frequently reported complications are pain, swelling, and difficulty opening the mouth during the post-operative period. As documented in the literature, various complications can occur during extractions, including mandibular fractures, nerve damage, and displacement of the tooth or its fragments into facial spaces [1]. The most serious surgical complications involve damage to the inferior alveolar nerve (IAN) and lingual nerve (LN), potentially leading to permanent neurosensory disturbances, with an incidence of 0.35% and 0.69%, respectively [2]. Another possible complication of lower wisdom tooth extraction, which may cause secondary LN damage, is the displacement of the tooth into various anatomical spaces, including parapharyngeal, pterygomandibular, sublingual, and submandibular spaces. Displacements into the submandibular space are the most common [3]. This displacement typically occurs due to excessive pressure, insufficient clinical and radiographic evaluations, an inappropriate surgical approach, and/or the patient’s unique anatomical features [4,5]. Depending on the clinical presentation, treatment may involve either removal of the displaced tooth or conservative management, with symptoms ranging from asymptomatic cases to reports of pain, swelling, and mandibular trismus [6]. Open surgical techniques, combined with proper pressure application and placement of retractors in the lingual region, can help minimize the occurrence of such complications [7,8]. Like many various phases of dental diagnostics and procedures [9,10,11,12], these types of complications can trigger medicolegal disputes that underscore the importance of experience in managing such events. Proper pre-operative clinical and radiographic planning can significantly reduce the risk of nerve sensitivity loss in oral and maxillofacial surgery.

While nerve sensitivity loss has a relatively low incidence in routine and oral maxillofacial surgeries [13], if symptoms occur, there are pharmacological protocols that can facilitate nerve recovery [14]. In cases of more extensive damage, such as nerve transection, microsurgical techniques are required to restore nerve integrity [15]. Fortunately, today’s surgeons have a wide range of diagnostic and surgical tools that greatly reduce the likelihood of such damage. In complex cases, three-dimensional imaging and planning allow for more accurate surgical steps that can minimize potential complications [16]. For example, in orthognathic surgery, cutting guides designed in a digital CAD/CAM environment can align surgical instruments so they avoid damaging critical structures. Similar advancements are seen in implant and orthodontic surgery, where guided techniques determine the angle and depth of implants or mini-screws in order to avoid nerve injury [17,18]. The literature on root migration into the submandibular space with permanent nerve damage is limited. The case report presented herein can fill the knowledge gap by providing insights into when and how to involve other specialists in managing and diagnosing complications. The two-stage surgical approach (initial extraction by an oral surgeon followed by root retrieval by a maxillofacial surgeon) is a distinctive aspect that highlights the need for a multidisciplinary approach in similar clinical situations. The permanence of neurological damage raises questions of professional liability and informed consent, which are relevant in the dental and surgical community. 

## 2. Case Report

In November 2023, a 23-year-old male patient came in for a dental evaluation. Before the clinical examination, the patient reported significant functional chewing impairments and a general sense of oral discomfort. He attributed these issues to complications that arose during the post-operative phase of an oral surgery procedure and subsequent maxillofacial re-surgery required for the extraction of the left lower third molar (i.e., tooth 38, according the FDI classification) [19].

### 2.1. Dental History

**Figure 1:**
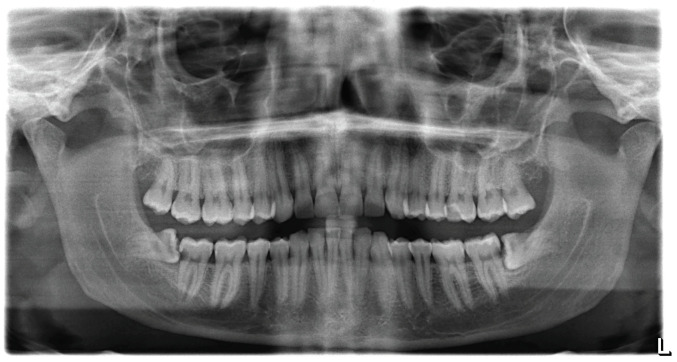
Initial panoramic radiograph of the patient with both lower wisdom teeth mesially inclined, partially impacted, and in strict contact with the distal wall of the adjacent tooth affected by dental caries.

**Figure 2:**
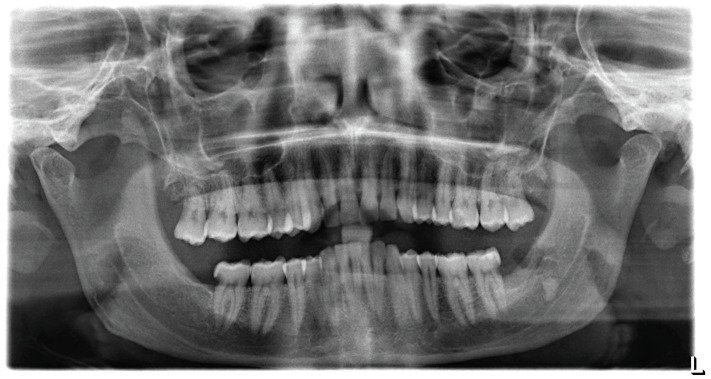
Panoramic radiograph taken after the lower wisdom teeth extraction, shown on the right side the residual fractured root.

**Figure 3:**
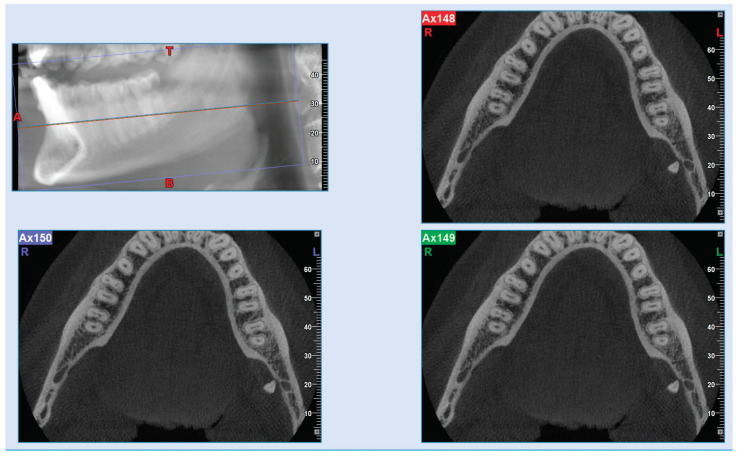
CBCT sagittal and axial views showing the position of the residual fractured root outside the bone in the submandibular space.

The patient showed up to our clinic with complaints following a wisdom tooth extraction. Periapical and panoramic radiographs (Figure 1) pointed to a mesially inclined and impacted tooth #38 having been recently extracted, which had caused caries in the distal portion of the adjacent tooth (tooth #37). A similar situation was observed on the opposite side; however, in that case, the wisdom tooth extraction had not led to any negative consequences. The extraction procedure was performed in March 2022, and lasted approximately two and a half hours. At the end of the procedure, the dentist took a post-operative dental radiograph (Figure 2) and informed the patient that a root fragment had not been removed. The dentist recommended a Cone-Beam CT scan to determine the fragment’s position. Following the dentist’s advice, the patient underwent a Cone-Beam CT scan at a medical center in March. On the same day, the patient returned to the dentist with the results, which showed that the root fragment had been displaced into the submandibular space (Figure 3), further necessitating a removal procedure at a hospital facility. The patient reported undergoing antibiotic and anti-inflammatory therapies for pain management and lowering the risk of infections prior to the surgery. In April 2022, the patient was admitted to the dentistry unit of a hospital where urgent maxillofacial surgery was deemed necessary. The patient was advised to consult a maxillofacial surgery specialist, who emphasized the importance of timely intervention to control the existing infection and prevent further complications caused by the dislocated root. Subsequently, the patient went to a private clinic, where a maxillofacial surgeon and their team performed the extraction of the dental root under both general and local anesthesia, as documented in the medical records provided to the patient. The procedure required a one-day hospital stay. Upon discharge, the patient was prescribed a supportive pharmacological therapy, including non-steroidal anti-inflammatory drugs (ibuprofen in a dosage of 400 mg every 4–6 h) and an antibiotic (amoxicillin with clavulanic acid in a dosage of 875 mg + 125 mg, twice daily for 5 days), as noted in the medical records.

### 2.2. Intra- and Extra-Oral Examination

Upon extra-oral objective examination, the patient exhibited a slight facial asymmetry on the left side, accompanied by hypotonia of the lower third of the face on the same side upon palpation. However, the mimic–postural dynamics of the perioral tissues were preserved. A detailed clinical assessment focused on muscle function, symmetry, and coordination. Initial observation of the perioral region at rest helped identify any asymmetries or muscle tensions, followed by an assessment of the overall head and neck posture to evaluate its influence on perioral tissue dynamics. Gentle palpation of the orbicularis oris and surrounding muscles, such as the zygomaticus and buccinator, was conducted to detect areas of tension, hypertonicity, or tenderness. Functional testing followed, where the patient was asked to make various facial expressions (i.e., smiling, frowning, puckering lips, tightly closing lips, and puffing out cheeks) to observe muscle activation, coordination, and any compensatory movements. The lip seal test assessed the patient’s ability to maintain lip closure against resistance, providing insight into muscle strength and endurance. Speech articulation was evaluated in terms of functional mobility and coordination of the lips. Changes in perioral muscle tone were observed when the patient changed their posture, helping identify postural patterns that influence the activity of mimic muscles. Normal tone was observed on the opposite side. The tongue was positioned at rest on the palate with no evident signs of lingual motor deficits or preserved papillae trophisms. Functional examination of the TMJ (temporo-mandibular joint) revealed no signs of TMD (temporo-mandibular disorder). The patient reported frequent sensations of masticatory muscle weakness (due to the impairement of mandibular movements involving the mylohyoid and anterior digastric muscles) and initial numbness (hypoesthesia/anesthesia) of the left hemimandible, including its mucosal and cutaneous attachments. The patient reported progression in paresthesia and dysesthesia. However, he maintained the actions of drinking and swallowing without deficits in the labial seal, with no involuntary saliva loss or accidental tongue biting. Neuropathic pain was not reported either. Upon inspection, it was observed that lip trophism was preserved despite sensory deficits, the vascular supply was unaffected, and there were no visible scars from accidental bites, burns, or other injuries.

### 2.3. Trigeminal Sensory Testing

Trigeminal sensory testing (NST) type A was performed including two-point discrimination, which appeared normal on both the affected side (left hemilabium) at the level of the hemilabium (vermilion) and the healthy side (right). Movement direction discrimination was altered on the affected side at the level of the skin, gums, and intraoral mucosa and was preserved on the healthy side (right hemimandible). Type B testing (light touch) showed altered results on the affected side and normal results on the healthy side. Thermal-stimulation testing (type C) revealed an altered response on the affected side compared to the healthy side. Testing with sharp and blunt instruments (type C) produced an altered responses on the affected side compared to the healthy side. While the healthy side showed normal responses to all stimuli, the affected side displayed reduced perception, which patient described as either dull or diminished.

### 2.4. Radiographic Documentation

Dental radiographs and cone-beam computer tomography (CBCT) revealed a dislocation, of what was likely the mesial root of a tooth, into the left submandibular space. No other pathologies related to current symptoms were evident and there were no lesions requiring dental intervention.

### 2.5. Electromyography, Somatosensory Evoked Potential (SEP), and Neurologist’s Opinion

A neurological examination with electromyography (EMG) and somatosensory evoked potential (SEP) of trigeminal masticatory muscles was recommended. The patient underwent a neurological examination with EMG and SEP in November 2023. Physical examination revealed hypoesthesia of the left V3 trigeminal territory. The electromyographic examination showed clinical and electrophysiological signs of sensory and motor neurogenic dysfunction in the left trigeminal nerve, with no signs of ongoing denervation. Although hypoesthesia of the left V3 trigeminal territory cannot be directly detected by EMG, motor dysfunction in V3-innervated muscles was evident. SEP testing, which is more appropriate for sensory evaluation, involved stimulation of the mental nerve and mandibular foramen, with recordings being made over the somatosensory cortex (C3/C4 or Cz). EMG electrodes were placed on the masseter and temporalis muscles to assess motor deficits. A follow-up specialist neurological examination in January 2024 confirmed the presence of permanent neurogenic impairment of the lower third branch of the left trigeminal nerve secondary to the extraction of the left wisdom tooth with subsequent maxillofacial surgery required for the removal of the dislocated root in the submandibular area. This led to severe neuropathy characterized by persistent hypoesthesia, which affects the inferior alveolar nerve.

### 2.6. Medicolegal Considerations

In this case, the patient who initially arrived for an in-depth diagnostic evaluation and possible resolution of his existing dental problem, later became the subject of a claim seeking compensatory damages and, hence, required medicolegal assistance. Medicolegal damage assessment and liability for the surgical injury highlighted the fact that the oral surgeon did not fully plan the operation, as they have failed to request comprehensive 3D diagnostic imaging in order to visualize the patient’s anatomical characteristics. This could have enabled a better risk assessment and could have possibly prevented the injury. However, the oral surgeon’s conduct in the post-operative phase, once the complication was recognized, was deemed ethically and professionally appropriate. Partial co-responsibility was attributed to the maxillofacial surgeon who, in removing the root from the submandibular space, may have worsened the situation. Nevertheless, this intervention was deemed necessary for preventing complications caused by the displacement of the dental fragment. The inferior alveolar nerve’s anatomical proximity to the submandibular space poses surgical challenges because its upper margin is closely associated with the medial surface of the mandible, which only partially separates it.

## 3. Discussion

Third molars often have varied eruption possibilities, are frequently impacted, and may require extraction due to their unfavorable positioning [20,21,22]. While third-molar dislocation is an undesirable complication, it is difficult to predict when a third molar is at risk [23]. Immediate retrieval of the dislocated fragment is not always possible, and requesting a CBCT scan is often the preferred course of action [24]. Sensory disorders of the inferior alveolar nerve can be evaluated early using magnetic resonance imaging (MRI) to assess signal intensity changes [25]. Early removal of the dislocated fragment is recommended because waiting may increase the risk of infection or foreign body reactions that vary individually [26,27]. In this case, the patient was appropriately managed during the emergency treatment. It is worth mentioning that dislocation of the lower third molar often occurs due to the thin lingual cortical bone of the mandible, which is influenced by individual anatomical factors [28]. Dislocation typically involves slipping beneath the mylohyoid muscle and into the submandibular space [29]. An evaluation conducted nearly eight months after the initial extraction and seven months after the re-intervention required to remove the root fragment revealed a more unfavorable prognosis, as expected [30]. A follow-up electromyographic (EMG) and SEP examination conducted at a later stage confirmed the presence of permanent damage [31]. The clinical and instrumental findings were consistent with a diagnosis of severe unilateral (left) inferior alveolar nerve neuropathy characterized by hypoesthesia [32,33]. Additionally, psychological damage was observed, although it was not directly evaluated in this study [34,35].

The most common sites for dislocation of impacted mandibular third-molar fragments are sublingual, submandibular, and pterygomandibular spaces. Attempts to retrieve fragments without proper visualization or surgical skills can lead to deeper displacement into these spaces [36,37]. In this case, the root was displaced into the submandibular space, which is located below the mylohyoid muscle and above the hyoid bone and platysma muscle [38]. The orientation of the root directly influences the risk of the mylohyoid nerve (a branch of the inferior alveolar nerve) injury [39]. Factors such as improper instrument use and individual anatomical characteristics further increase the risk of displacement, especially in cases of lingually positioned or deeply impacted teeth. Fractures or perforations of the lingual cortical bone, or its thin structure, combined with excessive pressure during extractions can cause dislocations into deeper anatomical areas [40]. Pre-operative evaluation of the risk of inferior alveolar nerve and/or lingual nerve injury requires radiographic imaging. The recent introduction of artificial intelligence (AI)-powered 3D models based on cone-beam computer tomography may improve risk assessment [41]. The role of AI in medicine has significant forensic implications [42,43] and could greatly benefit forensic investigations and medicolegal evaluations in dentistry [44,45,46,47,48]. A computer-assisted navigation system has recently been introduced into oral and maxillofacial surgery, allowing real-time 3D confirmation of the surgical site during procedures. This system helps prevent post-operative complications such as lingual nerve palsy [49,50]. In implantology, computer-assisted surgery does not require a steep learning curve [51], making it a valuable tool for performing safer third-molar extractions. Beyond pre-extraction diagnostic imaging, the integration of dynamic surgical guide systems could support both the oral and maxillofacial surgeon in this case, resulting in potentially improved outcomes.

Submandibular displacement of a tooth can lead to swelling, pain, restricted mandibular movement, severe tissue damage, psychological distress, and medicolegal complications [52,53]. It may also cause permanent or temporary neurological damage to the lingual nerve, resulting in paresthesia in the lateral region of the tongue, numbness, severe pain, burning sensations, and taste disturbances [54]. A case of a submasseteric abscess occurring one month after the displacement of an impacted lower third molar has been documented [55]. Before surgically removing a displaced root, developing a comprehensive treatment strategy is important for preventing complications. Some surgeons may delay surgery for a few weeks to allow fibrosis to anchor the tooth more securely [55]. However, postponing extraction for more than 24 h can cause inflammation, pain, swelling, trismus, infection, and further displacement or fracture of the root. An untreated tooth fragment left in place for a long period increases the risk of foreign body reactions and infections in neck spaces. Infections can also spread to cranial nerves (from IX to XII), erode the carotid artery, and cause internal jugular vein thrombosis [56]. If attempts to retrieve the displaced tooth are unsuccessful, it is recommended to immediately stop the intervention and refer the patient to a maxillofacial surgeon to prevent deeper displacements [57].

This clinical case highlights the importance of providing clear and informed consent regarding the risks of third-molar extraction to patients, especially to address potential legal disputes [58]. The use of advanced technologies, such as artificial intelligence, could guide clinicians in reducing diagnostic and operational errors [42,45,48,49]. In case of a tooth displacement into anatomical spaces, the dentist should inform the patient and provide relevant information to avoid delays and progression to dangerous conditions such as cervicofacial infection or Ludwig’s angina [35,51,52]. Functional examination and trigeminal sensory tests should also be conducted [59]. The literature suggests that the risk of complications related to third-molar displacement into adjacent anatomical spaces does not significantly differ between general dentists, oral surgeons, or maxillofacial surgeons [60]. However, when complications arise, dentists and oral surgeons typically refer patients to maxillofacial surgeons rather than managing the complication themselves, even if their perceived difficulty of the extraction is similar [61,62]. Globally, oral surgeons and maxillofacial surgeons are regarded similarly in terms of professional liability for third-molar extractions and complications, as seen in this case. This suggests that complications in shared anatomical spaces result in overlapping responsibilities between the two specialties [63,64,65,66,67]. Preventive measures, such as early radiographic monitoring of third molars around the age of 15 or 16 (when root development is minimal) could minimize complications by allowing safer extractions [22,68].

Rehabilitation of permanent inferior alveolar nerve damage is aimed at restoring its function and alleviating sensory disturbances. Recent therapeutic strategies include photobiomodulation therapy, microsurgical nerve repair, and pharmacological management [69,70,71]. A multidisciplinary approach is essential for improving outcomes in permanent nerve damage. In this case, the patient was treated with anti-inflammatory medications to manage early neuropathic pain. The absence of additional therapeutic interventions could be considered a limitation, as combined therapies might have improved the state of nerve injury. Future protocols should focus on managing these complications and enhancing nerve recovery.

## 4. Conclusions

Although the extraction of mandibular third molars is routine, complications can arise as pre-operative planning does not adequately account for anatomical variations. Displacement of tooth fragments into adjacent anatomical spaces may not always result in severe complications and nerve damage. However, in some cases, permanent damage can occur. Even without visible esthetic impairment, permanent sensory deficits significantly impact the patient’s quality of life, compounded by unfulfilled expectations of recovery. In this case, the involvement of two surgeons (i.e., the oral surgeon who performed the initial extraction, and the maxillofacial surgeon responsible for the recovery of displaced fragments) raises complex liability questions. Inadequate informed consent, deviation from standard surgical protocols, lacking pre-operative diagnosis, or mismanagement can lead to negligence-based malpractice claims. Comprehensive documentation and timely interventions are key factors for mitigating legal risks. Permanent sensory deficits may result in compensation claims for both functional and psychological impairments. Adhering to best surgical practices and ensuring clear communication are critical to reducing medicolegal exposure.

## Data Availability

No additional data are available.
